# Phylogenetic and Evolutionary Analysis of Chinese *Leishmania* Isolates Based on Multilocus Sequence Typing

**DOI:** 10.1371/journal.pone.0063124

**Published:** 2013-04-30

**Authors:** Chun-Ying Zhang, Xiao-Jun Lu, Xiao-Qing Du, Jun Jian, Ling Shu, Ying Ma

**Affiliations:** Division of Clinical Microbiology, Department of Laboratory Medicine, West China Hospital, Sichuan University, Chengdu, People’s Republic of China; Technion - Israel Institute of Technology, Israel

## Abstract

Leishmaniasis is a debilitating infectious disease that has a variety of clinical forms. In China, visceral leishmaniasis (VL) is the most common symptom, and *L. donovani* and/or *L. infantum* are the likely pathogens. In this study, multilocus sequence typing (MLST) of five enzyme-coding genes (*fh, g6pdh, icd, mpi, pgd*) and two conserved genes (*hsp70*, *lack*) was used to investigate the phylogenetic relationships of Chinese *Leishmania* strains. Concatenated alignment of the nucleotide sequences of the seven genes was analyzed and phylogenetic trees were constructed using neighbor-joining and maximum parsimony models. A set of additional sequences from 25 strains (24 strains belong to the *L. donovani* complex and one strain belongs to *L. gerbilli*) were retrieved from GenBank to infer the molecular evolutionary history of *Leishmania* from China and other endemic areas worldwide. Phylogenetic analyses consolidated Chinese *Leishmania* into four groups: (i) one clade A population comprised 13 isolates from different foci in China, which were pathogenic to humans and canines. This population was subdivided into two subclades, clade A1 and clade A2, which comprised sister organisms to the remaining members of the worldwide *L. donovani* complex; (ii) a population in clade B consisted of one reference strain of *L. turanica* and five Chinese strains from Xinjiang; (iii) clade C (SELF-7 and EJNI-154) formed a population that was closely related to clade B, and both isolates were identified as *L. gerbilli*; and (iv) the final group, clade D, included *Sauroleishmania* (LIZRD and KXG-E) and was distinct from the other strains. We hypothesize that the phylogeny of Chinese *Leishmania* is associated with the geographical origins rather than with the clinical forms (VL or CL) of leishmaniasis. To conclude, this study provides further molecular information on Chinese *Leishmania* isolates and the Chinese isolates appear to have a more complex evolutionary history than previously thought.

## Introduction

Leishmaniasis, a geographically widespread disease, is caused by infection with protozoan parasites of the genus *Leishmania*. The clinical forms are grouped into localized skin lesions of cutaneous leishmaniasis (CL), destructive metastatic nasopharyngeal lesions of mucocutaneous leishmaniasis (MCL), and progressive systemic visceral leishmaniasis (VL), which is fatal if left untreated [1]. The incidence of leishmaniasis is increasing, approximately 0.2 to 0.4 million VL cases and 0.7 to 1.2 million CL cases occur each year, with an estimate of 20,000 to 40,000 deaths as a result of leishmaniasis each year [2]. In recent years, *Leishmania*-HIV coinfection has been described as an emerging disease [3].

Leishmaniasis is also found in China, especially in the west frontier regions [4]–[6]. According to previous studies, *L. donovani* and/or *L. infantum* are the most common etiological pathogens in China [7], [8]. A national control program has largely brought the disease under control in eastern China, while leishmaniasis is considered to be endemic or with sporadic outbreaks in western China, including Xinjiang Uygur Autonomous Region, Gansu, Sichuan and Inner Mongolia [9], [10]. In 2008–2009, following an outbreak of the desert subtype of zoonotic VL, the number of infant VL infections dramatically increased in Jiashi County, Xinjiang [11].

The *Leishmania* species in China are complex. Various clinical forms exist, ranging from cutaneous to fatal VL, and they are caused by human/canine pathogenic *Leishmania* species. Some species infect gerbils and lizards, but these are nonpathogenic to humans [12]. According to different epidemiological characteristics, leishmaniasis in China has been classified into three types [13]: plain, hill and desert foci. The different hosts and vectors for the parasites in distinct foci make unveiling the relationship between *Leishmania* strains difficult. For example, *L. donovani* is generally considered to be the pathogen of plain type leishmaniasis, and only sporadic cases of this infection have been reported since the 1980s. In Gansu and Sichuan provinces, hill type cases of leishmaniasis are prevalent, and most patients are children aged less than 10 years. In this instance, domestic dogs are the key reservoir. Xinjiang is a mixed area with two epidemiological types, plain and desert [9], [11]. The plain type is endemic in the oases of the plains of Kashgar City, where children aged less than 5 years are susceptible. The desert type is distributed in the desert regions of Xinjiang. Two species were found in this area, one is *L. donovani* complex that causes autochthonous kala-azar in infants, and the other species is *L. turanica*, which is nonpathogenic to humans.

Research groups have investigated the phylogenetic relationship of the Chinese *Leishmania* strains using internal transcribed spacer 1 (ITS1) sequencing [14] and kinetoplast cytochrome oxidase II (COII) gene sequencing [15]. Some studies have classified Chinese isolates as *L. infantum* [16], [17], while other research groups suggest that Chinese *Leishmania* isolates do not form a monophyletic group (such as *L. donovani* or *L. infantum*) due to the presence of a novel, undescribed species of *Leishmania* [14], [15], [18]. However, most of these studies included single species isolates or isolates from a limit number of geographic regions. Therefore, the taxonomic diversity and phylogenetic relationship of Chinese *Leishmania* strains remain unclear.

As morphological distinction of *Leishmania* species is impractical, the immunological and DNA-based criteria are used to identify the different strains. The universally accepted standard procedure for characterizing and identifying strains of *Leishmania* is multilocus enzyme electrophoresis (MLEE) [19]. However, the drawbacks of MLEE have restricted its usage [20], [21] and this method has been challenged by many alternative methods for species discrimination using molecular markers. These include restriction fragment length polymorphism (RFLP) analysis of the specific gene or gene fragment, such as cpB/gp63 [22], multilocus microsatellite typing (MLMT) fragment analysis [16], [17], [23], and multilocus sequencing typing (MLST) which targets conserved genes [24], [25].

The introduction of molecular approaches, particularly the numerous typing methodologies with various targets, has called for a revision of *Leishmania* taxonomy [26], [27]. Multilocus sequence typing (MLST) have been used to provide new insights into the population genetics, taxonomy and evolutionary history of *Leishmania*, and multiple specific MLST targets are available for *Leishmania* species [24], [25], [28].

Based on the published targets for the sub-genus of *Leishmania*, we combined five enzyme-coding genes (*fh*, *g6pdh*, *icd*, *mpi* and *pgd*) and two conserved genes (*hsp70* and *lack*) to differentiate the Chinese *Leishmania* isolates and to investigate their phylogenetic relationships.

## Materials and Methods

### Ethics Statement

**Figure 1:**
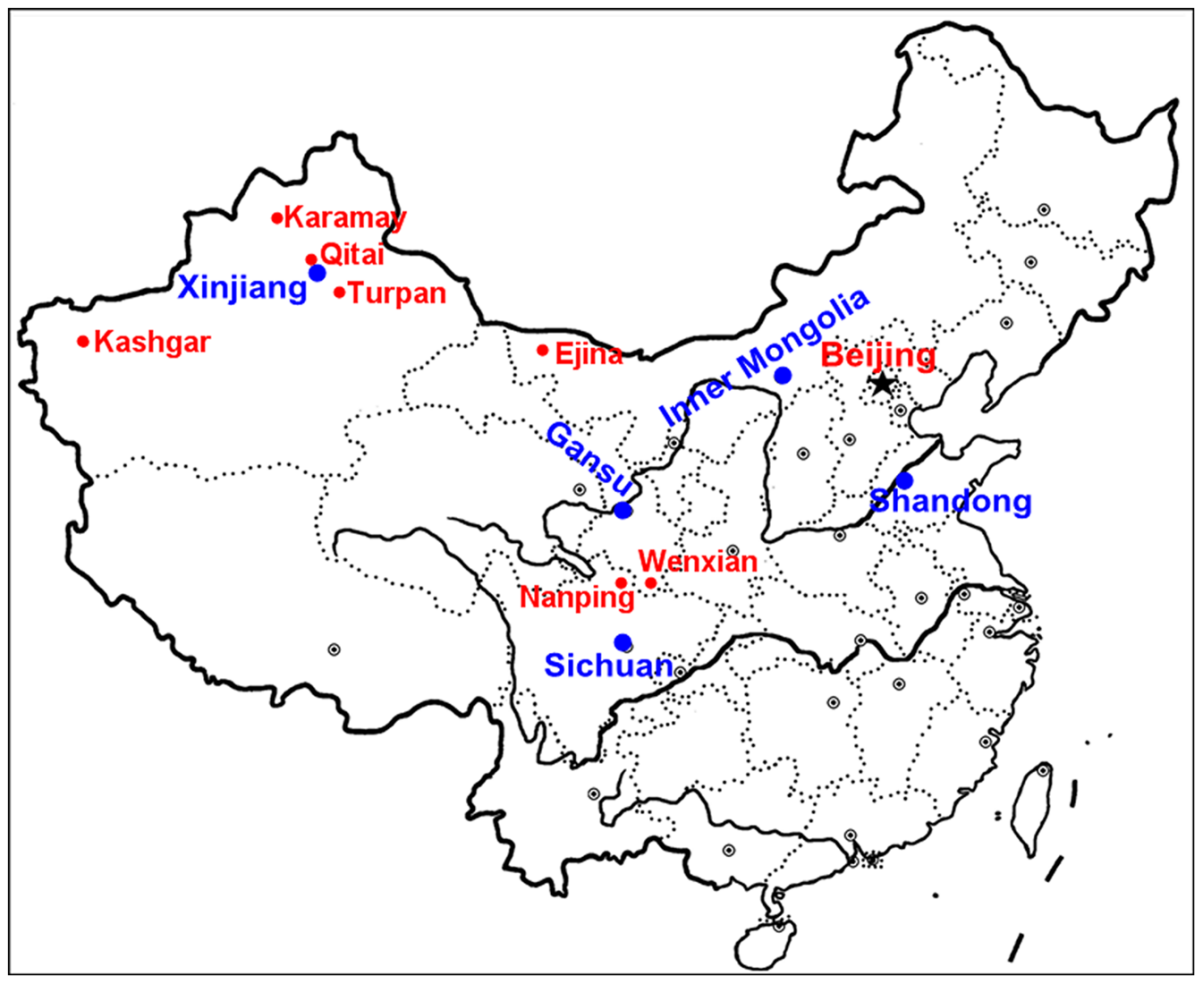
Locations of endemic areas in Xinjiang, Gansu, Sichuan, Inner Mongolia and Shandong regions of China. The *Leishmania* strains and their geographical origins are as follows: Xinjiang: Karamay (KXG-65, KXG-LIU, KXG-XU, KXG-Y, KXG-R, KXG-57, KXG-11, KXG-E); Kashgar (771, KS6, 801); Qitai (QITAI-15) and Turpan (SELF-7); Gansu: Wenxian (8801, WDD23) and (1101, 1102); Sichuan: Nanping (SC6, SC9); Inner Mongolia: Ejina (EJNI-154, LIZRD); Shandong: (9044).

Details of the WHO codes, sources, geographical origins and clinical manifestations of the 28 *Leishmania* isolates used in this study are listed in Table 1. Fig. 1 shows the different locations from where the Chinese strains had been isolated. We included 22 *Leishmania* strains isolated from different foci in China and 6 WHO *Leishmania* reference strains. Two strains, MCAN/CN/11/1101 and MCAN/CN/11/1102, were isolated from two dogs in China in 2011. The other 26 strains included in this study were kindly provided by Professor Wang Junyun at the Shanghai Municipal Center for Disease Control and Prevention, and has been previously analyzed by Wang Y et al. [29]. For the isolation of MCAN/CN/11/1101 and MCAN/CN/11/1102, oral informed consent was obtained from the guardians of the two dogs. The original animal work that produced the samples was conducted in accordance with guidelines of the Chinese Council on Animal Care, as approved by the Animal Care Committee of West China Hospital, Sichuan University.

**Table 1:** Details of the *Leishmania* isolates used in this study.

Name	Strain	Species	Location	Host	Disease	Accession numbers in GenBank						
						*fh*	*G6pdh*	*icd*	*mpi*	*pgd*	*Hsp70*	*lack*
(A) *Chinese strains and the WHO reference strains sequenced in this study*												
9044	MHOM/CN/90/9044		Shandong, China	Human	VL	JX021310	JX021329	JX021347	JX021369	JX021389	JX021428	JX021406
SC6	MHOM/CN/86/SC6		Nanping, Sichuan, China	Human	VL	JX021311	JX021330	JX021348	JX021370	JX021390	JX021429	JX021407
SC9	MCAN/CN/86/SC9		Nanping, Sichuan, China	Canine		JX021312	JX021331	JX021349	JX021371	JX021391	JX021430	JX021408
KXG-LIU	MHOM/CN/94/KXG-LIU	*L. infantum* a	Karamay, Xinjiang, China	Human	CL	JX021314	JX021333	JX021351	JX021373	JX021393	JX021432	JX021410
KXG-XU	MHOM/CN/93/KXG-XU	*L. infantum* a	Karamay, Xinjiang, China	Human	CL	JX021315	JX021334	JX021352	JX021374	JX021394	JX021433	JX021411
KXG-65	IMJW/CN/87/KXG-65		Karamay, Xinjiang, China	Sand fly		JX021318	JX021337	JX021355	JX021377	JX021397	JX021437	JX021415
771	IWUI/CN/77/771		Kashgar, Xinjiang, China	Sand fly		JX021307	JX021326	JX021344	JX021366	JX021386	JX021425	JX021403
801	MHOM/CN/80/801		Kashgar, Xinjiang, China	Human	VL	JX021323	JX021342	JX021361	JX021383	JX021402	JX970993	JX021422
KS6	MHOM/CN/96/KS6		Kashgar, Xinjiang, China	Human	VL	JX021324	JX970985	JX021364	JX021384	JX970991	JX970996	JX021423
8801	MHOM/CN/88/8801		Wenxian, Gansu, China	Human	VL	JX021325	JX021343	JX021365	JX021385	JX970992	JX970997	JX021424
1101	MCAN/CN/11/1101		Gansu, China	Canine		JX312703	JX312704	JX312706	JX312708	JX312709	JX312705	JX312707
1102	MCAN/CN/11/1102		Gansu, China	Canine		JX312710	JX312711	JX312713	JX312715	JX312716	JX312712	JX312714
WDD23	MCAN/CN/97/WDD23		Wenxian, Gansu, China	Canine		JX970981	JX970983	JX021362	JX970986	JX970989	JX970994	KC763807
KXG-Y	IMON/CN/90/KXG-Y	*L. turanica*	Karamay, Xinjiang, China	Sand fly		JX021308	JX021327	JX021345	JX021367	JX021387	JX021426	JX021304
KXG-R	IAND/CN/90/KXG-R	*L. turanica*	Karamay, Xinjiang, China	Sand fly		JX021319	JX021338	JX021356	JX021378	JX970988	JX021438	JX021416
KXG-57	MRHO/CN/90/KXG-57	*L. turanica*	Karamay, Xinjiang, China	Rodent		JX021320	JX021340	JX021358	JX021380	JX021399	JX021441	JX021419
QITAI-15	MRHO/CN/92/QITAI-15	*L. turanica*	Qitai, Xinjiang, China	Rodent		JX021321	JX021341	JX021359	JX021381	JX021400	JX021442	JX021420
KXG-11	MRHO/CN/87/KXG-11	*L. turanica*	Karamay, Xinjiang, China	Rodent		JX021322	JX970982	JX021360	JX021382	JX021401	JX021443	JX021421
SELF-7	IALE/CN/83/SELF-7	*L. gerbilli*	Turpan, Xinjiang, China	Sand fly		JX021313	JX021332	JX021350	JX021372	JX021392	JX021431	JX021409
EJNI-154	MRHO/CN/81/EJNI-154	*L. gerbilli*	Ejina, Inner Mongolia, China	Rodent		JX021316	JX021335	JX021353	JX021375	JX021395	JX021435	JX021413
LIZRD b	TRE/CN/80/LIZARD		Ejina, Inner Mongolia, China	Lizard							JX021434	JX021412
KXG-E b	IARP/CN/90/KXG-E		Karamay, Xinjiang, China	Sand fly							JX021439	JX021417
DD8 c	MHOM/IN/80/DD8	*L. donovani*	Bihar, India	Human	VL							KC763808
3720 c	MRHO/SU/80/CLONE3720	*L. turanica*	Uzbekistan	Rodent								KC763813
L100 c	MHOM/ET/72/L100	*L. aethiopica*	Wollo, Ethiopia	Human	CL							KC763812
K-27 c	MHOM/SU/74/K27	*L. tropica*	Azerbaijan	Human	CL							KC763809
5ASKH c	MHOM/SU/73/5ASKH	*L. major*	Turkmenistan	Human	CL							KC763810
M2903 c	MHOM/BR/75/M2903	*L. braziliensis*	Brazil	Human	CL							KC763811
(B) T*he strains whose sequences were taken from GenBank* d.												
LEM75	MHOM/FR/1978/LEM75		France	Human	VL	DQ449802	DQ449770	DQ449672	DQ449737	AM157139		
LPN114	MHOM/FR/1995/LPN114		France	Human	VL	DQ449803	DQ449771	DQ449673	DQ449738	AM157140		
PM1	MHOM/ES/1993/PM1		Spain	Human	VL	DQ449804	DQ449772	DQ449674	DQ449739	AM157141		
LSL29	MHOM/FR/1997/LSL29		France	Human	CL	DQ449805	DQ449773	DQ449675	DQ449740	AM157142		
BCN16	MHOM/ES/1986/BCN16		Spain	Human	CL	DQ449806	DQ449774	DQ449676	DQ449741	AM157143		
IMT260	MHOM/PT/2000/IMT260		Portugal	Human	CL	DQ449807	DQ449775	DQ449677	DQ449742	AM157144		
LEM3249	MHOM/FR/1996/LEM3249		France	Human	CL	DQ449808	DQ449776	DQ449678	DQ449743	AM157145		
LEM2298	MHOM/ES/1991/LEM2298		Spain	Human	VL	DQ449809	DQ449777	DQ449679	DQ449744	AM157146		
THAK35	MHOM/IN/1996/THAK35		India	Human	VL	DQ449811	DQ449779	DQ449681	DQ449746	AM157148		
GEBRE1	MHOM/ET/1972/GEBRE 1		Ethiopia	Human	VL	DQ449812	DQ449780	DQ449682	DQ449747	AM157736		
GILANI	MHOM/SD/1982/GILANI		Sudan	Human	VL	DQ449813	DQ449781	DQ449683	DQ449748	AM157149		
HUSSEN	MHOM/ET/0000/HUSSEN		Ethiopia	Human	VL	DQ449814	DQ449782	DQ449684	DQ449749	AM157150		
LEM189	MHOM/FR/1980/LEM189		France	Human	VL	DQ449815	DQ449783	DQ449685	DQ449750	AM157151		
BUCK	MHOM/MT/1985/BUCK		Malta	Human	VL	DQ449816	DQ449784	DQ449686	DQ449751	AM157152		
SC23	MHOM/IN/54/SC23		India	Human	VL	DQ449817	DQ449785	DQ449687	DQ449752	AM157153		
LEM3946	MCAN/SD/2000/LEM3946		Sudan	Canine	VL	DQ449818	DQ449786	DQ449688	DQ449753	AM157154		
3S	MHOM/SD/62/3S		Sudan	Human	VL	DQ449819	DQ449787	DQ449689	DQ449754	AM157155		
LLM175	MHOM/ES/88/LLM175		Spain	Human	VL	DQ449820	DQ449788	DQ449690	DQ449755	AM157156		
LLM373	MHOM/ES/92/LLM373		Spain	Human	VL	DQ449821	DQ449789	DQ449691	DQ449756	AM157157		
ISS1036	MHOM/IT/94/ISS1036		Italy	Human	VL	DQ449822	DQ449790	DQ449692	DQ449757	AM157158		
ISS800	MHOM/IT/93/ISS800		Italy	Human	VL	DQ449823	DQ449791	DQ449693	DQ449758	AM157159		
LEM3472	MHOM/SD/97/LEM3472		Sudan	Human	VL	DQ449824	DQ449792	DQ449694	DQ449759	AM157160		
LEM3429	MHOM/SD/97/LEM3429		Sudan	Human	VL	DQ449825	DQ449793	DQ449695	DQ449760	AM157161		
LEM3463	MHOM/SD/97/LEM3463		Sudan	Human	VL	DQ449826	DQ449794	DQ449696	DQ449761	AM157162		
Gerbilli	MRHO/CN/1960/Gerbilli	*L.gerbilli*		Rodent			DQ449800	DQ449699	DQ449767			

a The species identification is disputed dependent on the technique applied.

b Two strains LIZRD and KXG-E did not amplify the five enzyme-coding genes (*fh*?*g6pdh*?*icd*?*mpi* and *pgd*), so there is no accession numbers in GenBank.

c For the 6 WHO reference strains, we did not submit the corresponding sequences obtained here to GenBank because the sequences have been studied and submitted to GenBank by other researchers. Meanwhile, we aligned the sequences of the enzyme-coding genes and *hsp70* gene for the 6 reference strains we determined with those sequences retrieved from GenBank, the sequences for the genes we obtained were the same with those in GenBank, respectively.

d Strains whose sequences taken from GenBank were studied using MLST by Mauricio et al., 2006, and Zemanova et al., 2007, there are no sequences of *hsp70* and *lack* genes from the corresponding strains.

### Parasite Culture and Genomic DNA Preparation

The parasites were isolated as promastigotes on Novy-MacNeal-Nicolle biphasic culture medium and grown in Medium 199 supplemented with 15∼20% heat-inactivated fetal bovine serum at 22∼24°C. The promastigotes were harvested at room temperature by centrifugation at 4000 rpm for 10 min and washed three times with NET buffer (50 mM Tris-HCl, 125 mM EDTA, 50 mM NaCl). Total genomic DNA was extracted immediately using a proteinase K-phenol/chloroform method [30]. DNA was suspended in TE buffer (10 mM Tris-HCl, 1 mM EDTA) and stored at –20°C until required.

### PCR Amplification and Gene Sequencing

Five enzyme-coding genes [24], [25] (fumarate hydratase [*fh*], glucose-6-phosphate dehydrogenase [*g6pdh*], isocitrate dehydrogenase [*icd*], mannose phosphate isomerase [*mpi*], and 6-phosphogluconate dehydrogenase [*pgd*]), and two conserved genes [31], [32] (heat shock protein 70 [*hsp70*] and *Leishmania* homolog of receptors for activated protein kinase C [*lack*]) were used in this study. The primers were listed in Table 2. After an initial denaturation step for 10 min at 95°C, DNA samples were processed through 30 cycles of 1 min at 95°C, 1 min at the annealing temperature (T*_A_*) indicated in Table 2, 90 s at 72°C, followed by a terminal elongation step of 10 min at 72°C. Amplification products were examined by electrophoresis on 1.5% agarose gels and stained with ethidium bromide.

**Table 2:** Primers used for gene sequencing and PCR.

Target	Primer name a	Primer sequence	Y (*T_A_*)°C	Expected length (bp)	Sequences for alignment(begin to end)	length of the sequences (bp)	G+C contents	Ts/Tv b	Reference
*fh*	fh-f	AGCGTCTTGTGTTTCCCA	60	1707	7–1677	1671	63%	3.65	Zemanova et al., 2007
	fh-r	GAGCCCGTGTAAGGAGGC							
*g6pdh*	g6pdh-f	ATGTCGGAAGAGCAGTCT	50	1689	64–1641	1578	55%	3.68	Zemanova et al., 2007
	g6pdh-r	TCACAGCTTATTCGAGGGAA							
*icd*	icd-f	ATGTTCCGCCATGTTTCGGC	55	1308	58–1260	1203	59.5%	3.73	Zemanova et al., 2007
	icd-r	TTACGCGCTCATCGCCTT							
*mpi*	mpi-f	ATGTCTGAGCTCGTAAAGCT	55	1266	58–1212	1155	61%	2.38	Zemanova et al., 2007
	mpi-r	CTACCTGTCGCTCAAGTC							
*pgd*	pgd-f	GAACGAATCCCTTATTCTCYATG	60	1440	40–1431	1392	61.5%	2.41	Mauricio et al., 2006
	pgd-r	GGAACCGGTTGAGCGGC							
*hsp70*	hsp70-f	GACGGTGCCTGCCTACTTCAA	60	1380	40–1317	1278	65%	1.36	Fraga et al., 2010
	hsp70-r	CCGCCCATGCTCTGGTACATC							
*lack*	Lack-f	ACCATGAACTACGAGGGTCACCT	41	942	49–897	849	66.2%	2.18	Gonzalez-Aseguinolaza
	Lack-r	TTACTCGGCGTCGGAGAT							et al., 1999

a f is forward and r is reverse.

b Ts/Tv, transition/transversion ratios.

PCR products of the expected size were sequenced directly in both strands by ABI 3730 DNA Sequencers (Applied Biosystems) using the same primer pairs in the initial PCR reactions. The sequences were assembled with the aid of ContigExpress (http://www.contigexpress.com/), and a consensus sequence was generated for each strain. Apart from strain WDD23 for *g6pdh*, no gaps or insertions was detected in the sequences obtained from the strains used in this study. The gene sequences were deposited in GenBank (accession numbers shown in Table 1).

### Nucleotide Sequence Analysis

The sequences were aligned using ClustalW program in the MEGA 5.0 package [33]. The aligned matrix from this procedure was manually rechecked and minor adjustments were made if required. The transition/transversion ratios were calculated using the Kimura-2-Parameter and the synonymous and nonsynonymous substitution rates of selection were calculated using the modified Nei-Gojobori method (Jukes-Cantor distance model) with the MEGA 5.0 package.

### Protein Sequence Analysis

Molecular weight, overall charge, and charge variation according to theoretical isoelectric point (*pI*) for each partial protein sequence were determined using online resources (http://web.expasy.org/compute_pi/).

### Phylogenetic Analysis

Phylogenetic trees were constructed with each single gene sequence of *fh*, *g6pdh*, *icd*, *mpi*, *pgd*, *hsp70* and *lack* with one or no heterozygous genes. The DNA sequences of strains with one or more heterozygous sites were deconstructed into two (or several) individual sequences. As the similarity in sequences produced poor resolution result of a single gene tree, the concatenated alignment of the nucleotide sequences for the five enzyme-coding genes or all seven genes were analyzed. Prior to phylogenetic analyses, the most appropriate model of evolution, GTR+I+G, was determined using jModelTest v.0.1.1 [34] under the Akaike Information Criterion (AIC). Different statistical methods (for example, the maximum likelihood tree, UPGMA tree and neighbor-joining tree) were calculated in MEGA 5.0 [33]. The support of monophyletic groups was assessed by the bootstrap method with 1,000 replicates. Simultaneously, the PAUP (version 4.0b) [35] program was used to construct phylogenetic trees by maximum parsimony analyses and to perform bootstrap with 1,000 replications.

A set of additional sequences of 25 strains (shown in Table 1, 24 strains belong to the *L. donovani* complex and one strain belong to *L. gerbilli*) were retrieved from GenBank for the global analyses. The retrieved sequences were from the studies of Mauricio *et al*. [24] and Zemanova *et al*. [25]. Alignment simulations were performed using the ClustalW program. Phylogenetic and molecular evolutionary analyses were conducted using MEGA 5.0 and PAUP 4.0b as mentioned above.

## Results

### PCR Amplification of the Seven Genes

Using the primers listed in Table 2, a single product of the expected size for each gene was amplified from each of the Chinese *Leishmania* strains and the WHO reference strains. Three strains (LIZRD, KXG-E and M2903) only yielded the gene sequences of *hsp70* and *lack*. As only the region between the primers was sequenced, only partial section of the entire coding region of each gene was analyzed. The lengths of the sequences, the nucleotide positions of the start and the end of the sequences analyzed for the genes, their G+C contents and the transition/transversion ratios (Ts/Tv) were given in Table 2. The nonsynonymous/synonymous substitution ratios (dN/dS) varied, though they were all below 1.00 for seven genes. On a Z-test for selection, all the genes were neutral.

### DNA Sequences

Two major groups of aligned sequences were noted. The first group included 13 Chinese isolates and a WHO *L. donovani* reference strain DD8. The second group included five Chinese isolates (strain KXG-Y, KXG-R, KXG-57, KXG-11 and QITAI-15) and a WHO *L. turanica* reference strain 3720 as reported in a previous study [29]. When the two groups were compared, several nucleotide differences at numerous sites were found, so the DNA sequences were analyzed for the two groups, respectively.

Table 3 shows the polymorphisms within the group of 13 Chinese isolates. The number of single nucleotide polymorphic (SNP) sites varied among the genes. For example, there were seven mutations in the 1671 bp (7/1671) section of the *fh* gene, of which four were silent and three resulted in altered amino acid residues. Six SNPs (6/1578) were observed in *g6pdh*, and no alternative amino acid was identified. Two mutations were detected in both the *icd* and *pgd* genes, no amino acid change was observed in *icd*, and one position was involved with an amino acid change in *pgd*. Five diversity nucleotides were displayed in *mpi*, with one change in an amino acid residue. A low level of polymorphism was revealed for *hsp70* among the isolates. There were three heterozygous sites for strain 801. A high degree of similarity was observed among the *lack* gene sequences, and no amino acid changes were detected.

**Table 3:** Gene polymorphisms within the clade A for Chinese *Leishmania* isolates and WHO reference strain MHOM/IN/80/DD8.

Gene	Strain	Nucleotide position/Nucleotide							Alelle	pI	MMass
		**71**	189	1071	1245	**1484**	**1643**	1668		6.34	61609.10
***fh***	DD8, SC6	C	C	C	C	G	A	T	1	6.34	61609.10
	771, 9044, KXG-LIU, KXG-XU	C	T	T	G	G	A	C	2	6.34	61609.10
	KXG-65	**T**	T	T	G	G	A	T	3	6.94	52725.10
	SC9	C	C	C	C	**A**	A	T	4	6.31	61486.88
	1101, 1102, 801, WDD23	C	C	C	C	**A**	A	T	4	6.34	61636.12
	KS6, 8801	C	C	C	C	**A**	**T**	T	5	6.44	61620.17
		303	423	522	1413	1580	1595	1596			
***g6pdh***	DD8	C	T	T	T	T	G	C	1	5.73	59771.14
	771, 9044, KXG-LIU, KXG-XU, KXG-65	C	C	T	C	T	G	C	2	5.73	59771.14
	SC6	C	C	C	C	T	G	C	3	5.73	59771.14
	SC9, 1101, 1102, 801, 8801	T	T	T	C	T	G	C	4	5.73	59771.14
	WDD23	T	T	T	C	**–**	G	C	5	5.65	59577.84
	KS6	T	T	T	C	T	C	C	6	5.73	59785.17
		315	894								
***icd***	DD8, 771, 9044, SC6, KXG-LIU, KXG-XU, KXG-65	C	C						1	7.72	45087.56
	SC9,1101, 1102, 801, WDD23, KS6, 8801	T	A						2	7.72	45087.56
		258	**499**	600	756	1059					
***mpi***	DD8,771,9044, KXG-LIU, KXG-XU	T	G	G	T	G			1	5.91	42727.90
	KXG-65	T	G	G	T	A			2	5.91	42727.90
	SC6	T	G	A	T	G			3	5.91	42727.90
	SC9, 1101, 1102, WDD23	C	**A**	G	C	G			4	6.18	42726.96
	801, KS6, 8801	C	G	G	C	G			5	5.91	42727.90
		840	**952**								
***pgd***	DD8	C	**A**						1	6.24	50858.08
	9044, KXG-LIU, KXG-XU, SC6	C	**G**						2	6.01	50859.06
	SC9, 1101, 1102	C	**G**						2	6.01	50859.06
	801, KS6, 8801, WDD23	C	**G**						2	6.01	50859.06
	771, KXG-65	A	**G**						3	6.01	50859.06
		385	**887**	**1010**	1161	**1193**					
***hsp70***	DD8,9044,SC6,KXG-LIU, KXG-XU	C	C	C	C	C			1	5.28	46951.92
	771	C	**C/T**	C	C	C			1/2	5.23	46814.78
	KXG-65	C	**T**	C	C	C			2	5.23	46814.78
	SC9, 1101, 1102,8801, WDD23, KS6	T	C	C	C	C			3	5.28	46951.92
	801	T	C	**C/G**	C/T	**C/A**			3/4	5.65	46806.92
		600	759								
***lack***	DD8	C	T						1	6.04	31206.98
	771, 9044, SC6,	T	C						2	6.04	31206.98
	SC6, KXG-LIU, KXG-XU	C	C						3	6.04	31206.98
	SC9, 1101, 1102, 801, WDD23, KS6, 8801	C	T						1	6.04	31206.98

* Nucleotide differences in sequences of individual genes for 14 strains in clade A are shown, along with the position of the base mutations.

In bold: sites that cause an amino acid polymorphism. See deposited sequences for additional details.

For the six strains (3720, KXG-Y, KXG-R, KXG-57, KXG-11 and QITAI-15) of *L. turanica* mentioned above, the sequences had a high degree of similarity, apart from the synonymous mutations that occurred at a few mutant sites.

### Phylogenetic Trees

**Figure 2:**
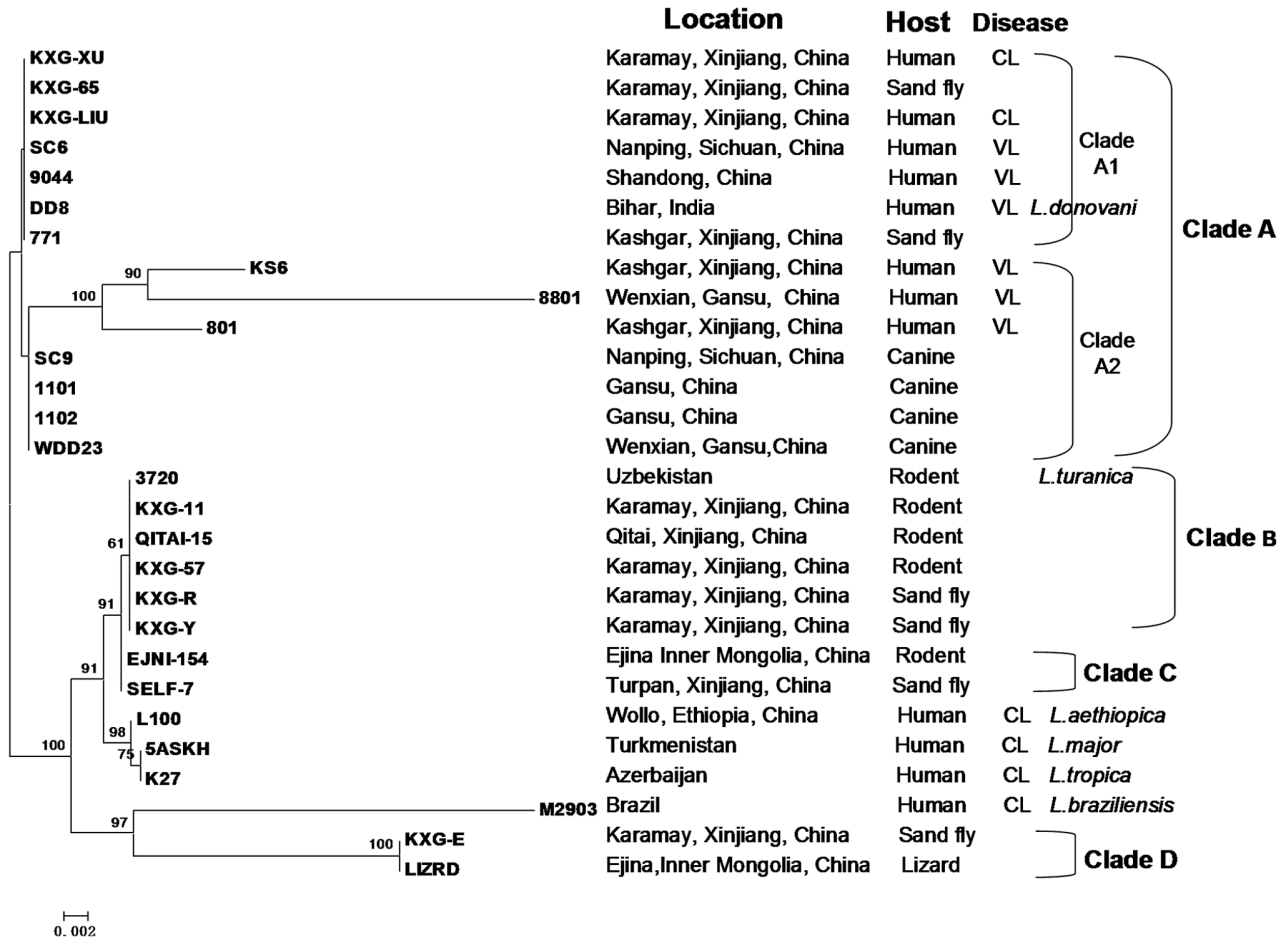
Neighbor-joining unrooted tree constructed from *hsp70* and *lack* genes for 28 isolates in this study. The Kimura-2-parameter method was used. Numbers above branches correspond to bootstrap valued based on 1,000 replicates. The strains were designated by their names (See Table 1 for more details).

The initial tree constructed based on individual gene comparisons did not produce discrepancy tree topologies (data not shown). The tree shown in Fig.2 was constructed based on the concatenated sequences of *hsp70* and *lack* using the neighbor-joining method in MEGA5.0 package. The phylogenetic analysis of *hsp70* and *lack* showed that 13 Chinese *Leishmania* isolates grouped as a population in clade A, which were well separated from other strains. Clade A was further divided into two subpopulations, clade A1 and clade A2. Five Chinese strains formed a group (clade B) with WHO *L. turanica* reference strain 3720, as reported previously [29]. Two distinct groups were also produced, one including SELF-7 and EJNI-154 (clade C) which were closely related to clade B in the tree, and the other population (clade D), consisting of LIZRD and KXG-E, was separated from the other Chinese isolates.

Phylogenetic tree was also constructed based on the concatenated sequences of the seven genes (Supplement Fig.1). Only 25 isolates were included in the analysis: three isolates, LIZRD, KXG-E and M2903, were excluded as only *hsp70* and *lack* genes were amplified. The tree topologies calculated by using different statistical methods or substitution models did not differ, so only the tree constructed by the neighbor-joining method based on Kimura 2-Parameter distance matrices in the MEGA 5.0 package (Supplement Fig.1) was presented, bootstraps with 1,000 replicates were applied. Three major clades (clades A, B and C) were also recognized in the neighbor-joining tree based on the combined sequences. Clade A included a WHO *L. donovani* reference strain DD8 and Chinese isolates which were pathogenic to humans and canines, and the Clade A can be subdivided into two general subclades: subclade A1 included the majority of strains from humans and sandflies; subclade A2 contained the strains isolated from humans and canines. The coexistence of different disease phenotypes (VL and CL) in the same species complex (clade A) was noted in this study, more strikingly, the same genotype from different disease phenotypes. In a previous study, incongruence between the *Leishmania* genotypes and the VL/CL disease phenotype of the isolates had also been noted [7]. Clade B included five Chinese *Leishmania* strains and a WHO reference *L. turanica* strain 3720. A distinct group, clade C, was detected for strain SELF-7 and EJNI-154, which were identified as *L. gerbilli*.

**Figure 3:**
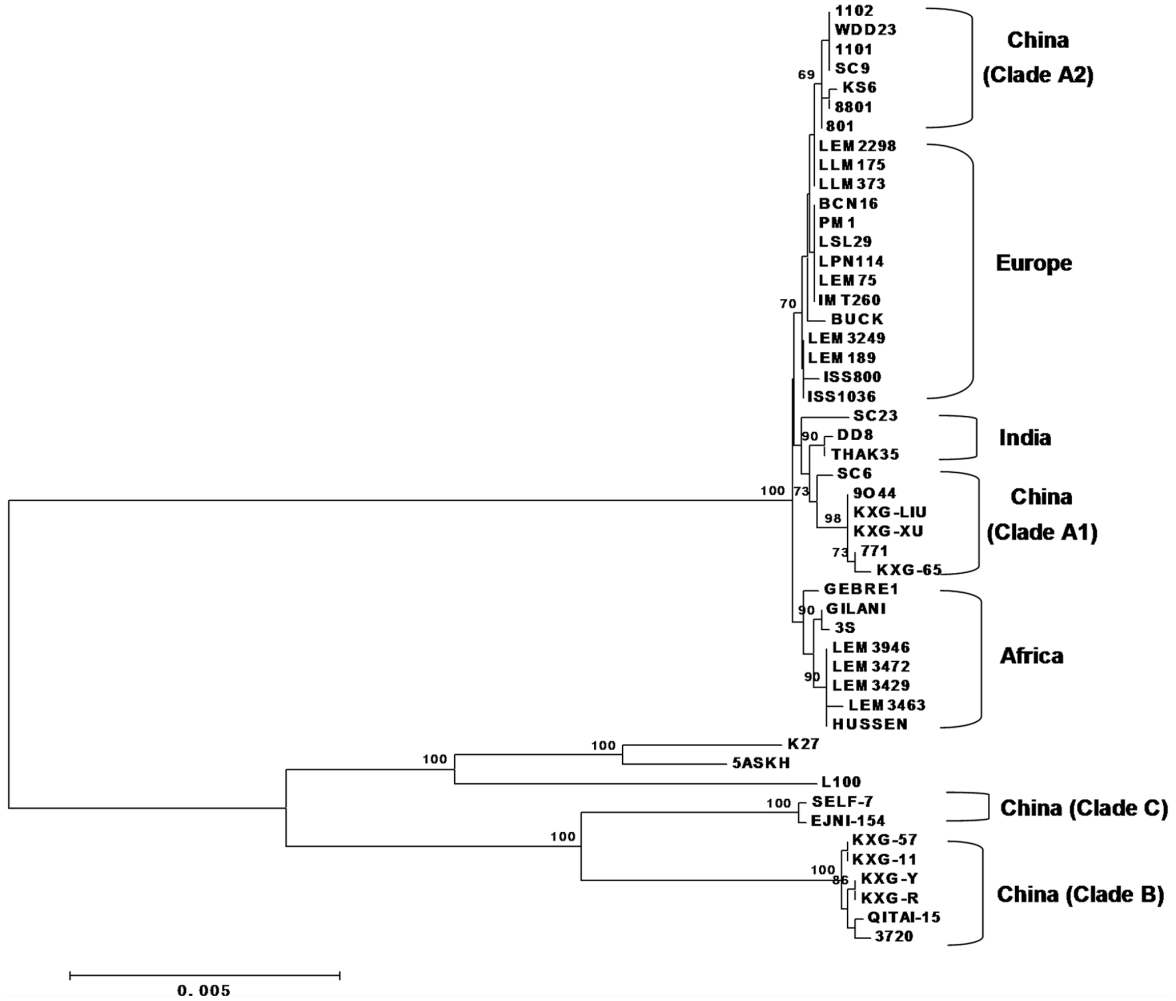
Phylogenetic trees constructed based on the sequences of the *fh*, *g6pdh* **,** *icd*, *mpi* and *pgd* genes for 25 isolates in this study and 24 isolates of the *Leishmanaia donovani* complex from other studies. Neighbor-joining tree was constructed from five enzyme genes for the 49 isolates using MEGA 5.0 software. The Kimura-2-parameter method was used. Numbers above branches correspond to bootstrap values based on 1,000 replicates. The strains were designated by their names (See Table 1 for more details).

We also investigated the phylogenetic relationships between Chinese *Leishmania* isolates in conjunction with isolates from other geographical regions. The trees generated by the entire dataset are displayed in Fig. 3 (and Supplement Fig.2). In both the neighbor-joining and maximum parsimony trees, 13 Chinese isolates formed two subclades which were separate from other strains of the *L. donovani* complex isolated from Europe, India and Africa. The branches that clustered in the tree reflected the geographical origins of the *Leishmania* strains. The six isolates from Xinjiang, Sichuan and Shandong Province in China comprised the subclade A1, the other seven isolates from Xinjiang, Sichuan and Gansu provinces in China supported the subclade A2. Additionally, the strains in clade A1 were close to the Indian and Africa isolates, while strains in clade A2 were close to the European isolates.

## Discussion

Multilocus sequence typing (MLST) has the potential to provide a substantial contribution to the understanding of the epidemiology, transmission and phylogenetics of infectious diseases, and the data can then be shared by depositing or withdrawing information from the GenBank, EMBL or DDBJ databases [36]. In this study, seven gene targets (*fh, g6pdh, icd, mpi, pgd, hsp70* and *lack*) were described for *Leishmania* strains in China, and the phylogenetic relationships and evolution of Chinese *Leishmania* isolates were investigated.

Despite the low level of SNP variation observed in our study, the existing differences of concatenated sequences reveal significant genetic diversity. The neighbor-joining or maximum parsimony methods identified several clusters when gene sequences of Chinese *Leishmania* strains were aligned with those of the WHO reference strains. In fact, these clusters were also observed in other tree topologies using different models (data not shown), which indicates that the derived groups were robust and not dependent on the choice of evolutionary methods underlying the various tree-building algorithms. Within the *Leishmania* subgenus, there was correlation between intraspecies divergence and the geographic distribution of the species. Within clade A, isolates from Shandong and Karamay of Xinjiang (clade A1) apparently differed from the majority of the closely related strains isolated from Gansu and Kashgar of Xinjiang (clade A2). This further demonstrated that the geographic origin of a strain was a more important predictor of genetic relationship than the type of disease presentation [37].

It has been proposed that environmental changes of human origin may be attributed to population structure and phylogenetic diversity [38]. The hosts play important roles in determining the outcome of leishmaniasis. The parasites in different hosts may develop different escape responses that result in variable mutation rates. Minor differences exist between strain SC6 from a patient and SC9 from a canine may have been a result of the host/reservoir origins. More isolates from humans and canines in Sichuan Province are needed to illustrate the phenomenon.

In clade B, five isolates from Xinjiang, an autonomous region of China, were highlighted by substantial genetic differentiations from *L. donovani* complex at the species level. The result of seven conserved genes indicated that the five strains were closely related to strain 3720, which was classified as *L. turanica*. Our results support the conclusion of previous studies [29] that these five strains were grouped as *L. turanica*. Two strains (SELF-7 and EJNI-154) in clade C have been previously identified as *L. gerbilli* [29]. The sequences of *g6pdh*, *icd* and *mpi* for strain SELF-7 and EJNI-154 were aligned with the WHO *L. gerbilli* reference strain, MRHO/CN/1960/Gerbilli, in the analysis, and strain SELF-7, EJNI-154 were corroborated as *L. gerbilli*. Additionally, five enzyme genes did not amplify from three isolates (LIZRD, KXG-E and M2903). Strain LIZRD and KXG-E were grouped into clade D in Fig. 2 when the *hsp70* and *lack* genes were analyzed. The strain KXG-E was genetically similar to *Sauroleishmania* strain LIZRD described by Wang *et al*. [29]. As *Sauroleishmania* and *Leishmania* are genetically significantly different [39], the changes in the primer annealing sites for strains LIZRD and KXG-E may have caused the failure in amplification. New primers located in the intragenic region of the corresponding enzyme-coding genes were designed and only part of the sequence for the single enzyme-coding gene was amplified. For strain M2903 of *L. braziliensis*, a New World species that belongs to a different sub-genus (*Viannia*), it was found to be distantly related to the Old World species. The enzyme-coding genes of this strain were not amplified with the primers used in this study as a result of the inappropriate primers.

Based on the trees constructed from our database and other sequences of the *L. donovani* complex worldwide, the results demonstrate that Chinese *Leishmania* isolates (clade A1 and A2) are sister to other members of the *L. donovani* complex. *L. donovani* complex species from China were segregated into two groups: clade A1 was most closely related to *L. donovani* strains from India and Africa, whereas clade A2 species were found to be related to the European strains of *L. infantum*. In the tree shown in Fig. 3, the clade A1 strains were placed between the *Leishmania* species from India and Africa. Previous finding has shown that Indian and African *L. donovani* strains differ considerably, and our results were in accordance with the previous study [40]. Thus, we further provide an evidence of a robust grouping of Chinese *Leishmania* when compared with the worldwide isolates, which provides a strong indication that the isolates from China have a more complex evolutionary history than previously thought. This study provides a basis for a further understanding of the phylogeny and evolutionary history of *Leishmania* parasitic species in China.

Whether Chinese *Leishmania* isolates are actually grouped as an undescribed species on the basis of new insights is a matter of debate. In the present study, sampling bias may exist for phylogenies, even though we tried to overcome this problem by selecting strains from diverse geographic origins and hosts. In addition, sequence variants may exist among different cells in a given population when uncloned cultures are used for DNA isolation. The direct DNA isolation of *Leishmania* from clinical samples would avoid this potential complication in the analysis. Further studies based on the current dataset of Chinese *Leishmania* isolates should be undertaken to gain further insight into the classification and evolution of *Leishmania* parasites. A systematic comparison of a statistically significant number of worldwide isolates would be an ideal way to elucidate the evolutionary profile of *Leishmania* species.

## Supporting Information

Figure S1 **Phylogenetic trees constructed based on sequences of the** ***fh*** **,** ***g6pdh*** **,** ***icd*** **,** ***mpi*** **,** ***pgd*** **,** ***hsp70*** **and** ***lack*** **genes for 25** ***Leishmania*** **isolates in this study.** The neighbor-joining unrooted tree was constructed using MEGA 5.0 software. The Kimura-2-parameter method was used. Numbers above branches correspond to bootstrap values based on 1,000 replicates. The strains were designated by their names (see Table 1 for more details).
(TIF)

Figure S2 **Phylogenetic trees constructed based on the sequences of the** ***fh*** **,** ***g6pdh*** **,** ***icd*** **,** ***mpi*** **and** ***pgd*** **genes for the 25 isolates in this study and 24 isolates of the** ***Leishmania donovani*** **complex from other studies.** Maximum parsimony tree constructed with the sequences of five enzyme genes for 49 isolates using the PAUP 4.0b program. The trees were rooted with *L. tropica* (K27) and *L. major* (5ASKH). Numbers above branches correspond to the bootstrap values based on 1,000 replicates. The strains were designated by their names (see Table 1 for more details).
(TIF)
