# Supplementary figures and images for: Phylogenetic and Evolutionary Analysis of Chinese Leishmania Isolates Based on Multilocus Sequence Typing
Source: PLoS One. 2013 Apr 30;8(4):e63124. doi: 10.1371/journal.pone.0063124 (PMC3639960; doi:10.1371/journal.pone.0063124)

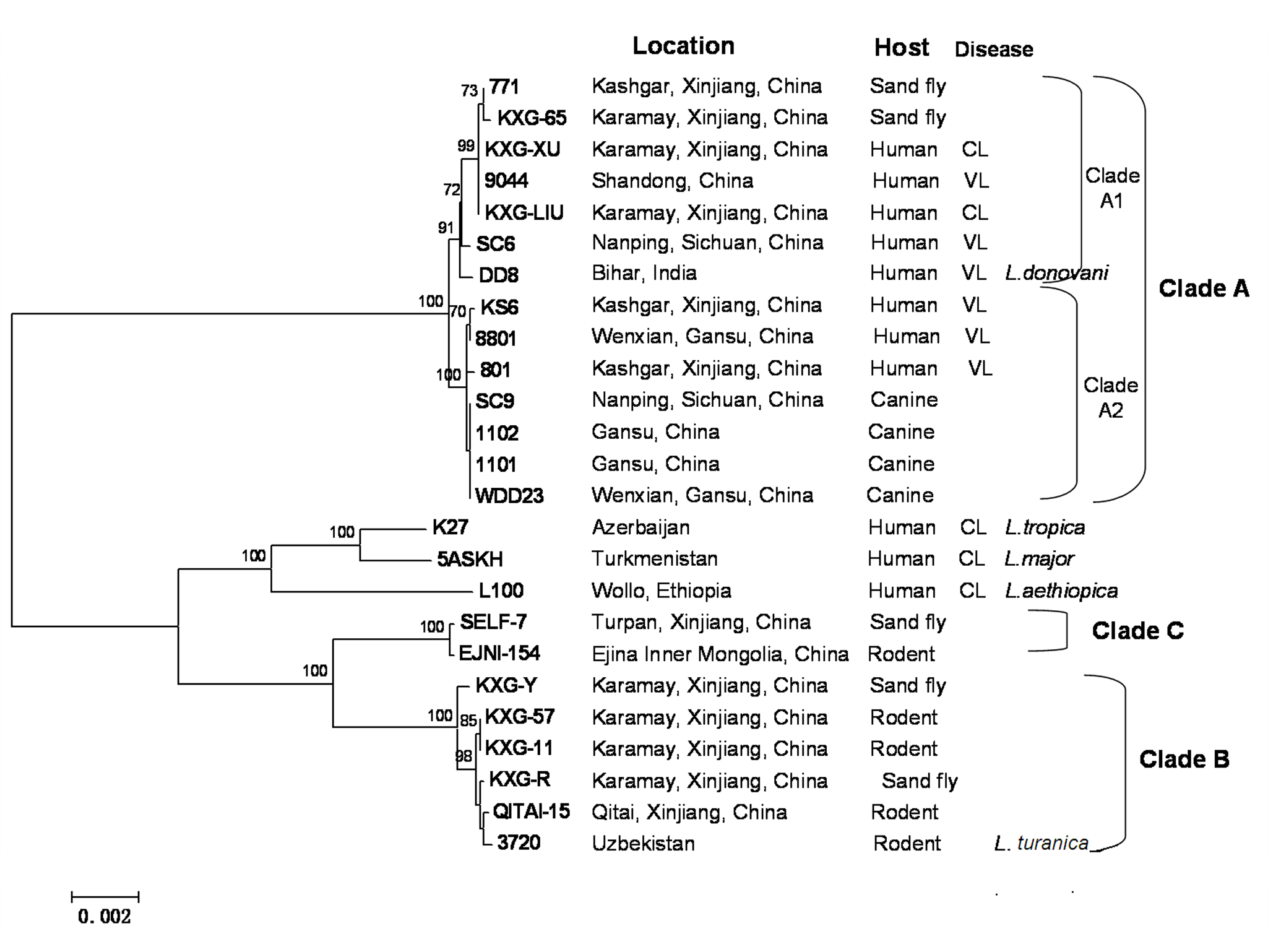

Supplement: Figure S1 — Phylogenetic trees constructed based on sequences of the fh , g6pdh , icd , mpi , pgd , hsp70 and lack genes for 25 Leishmania isolates in this study. The neighbor-joining unrooted tree was constructed using MEGA 5.0 software. The Kimura-2-parameter method was used. Numbers above branches correspond to bootstrap values based on 1,000 replicates. The strains were designated by their names (see Table 1 for more details). (TIF) [file pone.0063124.s001.tif]

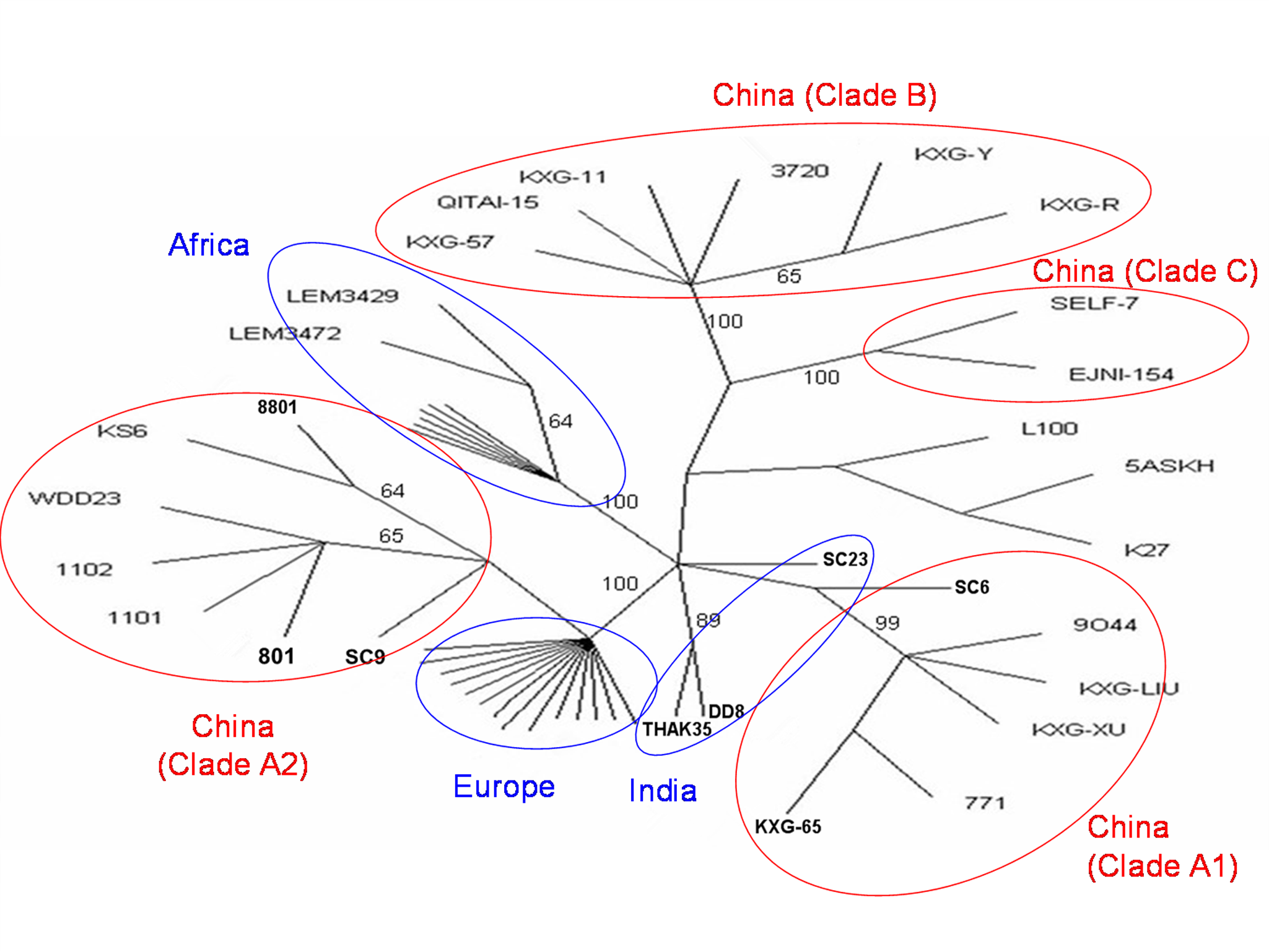

Supplement: Figure S2 — Phylogenetic trees constructed based on the sequences of the fh , g6pdh , icd , mpi and pgd genes for the 25 isolates in this study and 24 isolates of the Leishmania donovani complex from other studies. Maximum parsimony tree constructed with the sequences of five enzyme genes for 49 isolates using the PAUP 4.0b program. The trees were rooted with L. tropica (K27) and L. major (5ASKH). Numbers above branches correspond to the bootstrap values based on 1,000 replicates. The strains were designated by their names (see Table 1 for more details). (TIF) [file pone.0063124.s002.tif]
